# Construction of a novel cancer-associated fibroblast-related signature to predict clinical outcome and immune response in colon adenocarcinoma

**DOI:** 10.18632/aging.205032

**Published:** 2023-09-16

**Authors:** Lei Zheng, Jiale Zhang, Yingquan Ye, Zhangpeng Shi, Yi Huang, Mengmeng Zhang, Zhongxuan Gui, Ping Li, Huanlong Qin, Weijie Sun, Mei Zhang

**Affiliations:** 1 Department of Integrated Chinese and Western Medicine Oncology, The First Affiliated Hospital of Anhui Medical University, Hefei, China; 2 Department of Gastrointestinal Surgery, The First Affiliated Hospital of Anhui Medical University, Hefei, China; 3 Shanghai Clinical College, Anhui Medical University, Shanghai, China; 4 Department of General Surgery, Shanghai Tenth People’s Hospital, School of Medicine, Tongji University, Shanghai, China; 5 The Fifth Clinical Medical College of Anhui Medical University, Hefei, China; 6 Department of Infectious Diseases, The First Affiliated Hospital of Anhui Medical University, Hefei, China

**Keywords:** colon adenocarcinoma, cancer-associated fibroblasts, prognosis, immunotherapy, machine learning

## Abstract

The interaction between the tumour and the surrounding microenvironment determines the malignant biological behaviour of the tumour. Cancer-associated fibroblasts (CAFs) coordinate crosstalk between cancer cells in the tumour immune microenvironment (TIME) and are extensively involved in tumour malignant behaviours, such as immune evasion, invasion and drug resistance. Here, we performed differential and prognostic analyses of genes associated with CAFs and constructed CAF-related signatures (CAFRs) to predict clinical outcomes in individuals with colon adenocarcinoma (COAD) based on machine learning algorithms. The CAFRs were further validated in an external independent cohort, GSE17538. Additionally, Cox regression, receiver operating characteristic (ROC) and clinical correlation analysis were utilised to systematically assess the CAFRs. Moreover, CIBERSORT, single sample Gene Set Enrichment Analysis (ssGSEA) and Estimation of Stromal and Immune cells in MAlignant Tumor tissues using Expression data (ESTIMATE) analysis were utilised to characterise the TIME in patients with COAD. Microsatellite instability (MSI) and tumour mutation burden were also analysed. Furthermore, Gene Set Variation Analysis (GSVA), Kyoto Encyclopedia of Genes and Genomes (KEGG) and Gene Ontology (GO) elucidated the biological functions and signalling pathways involved in the CAFRs. Consensus clustering analysis was used for the immunological analysis of patients with COAD. Finally, the pRRophic algorithm was used for sensitivity analysis of common drugs. The CAFRs constructed herein can better predict the prognosis in COAD. The cluster analysis based on the CAFRs can effectively differentiate between immune ‘hot’ and ‘cold’ tumours, determine the beneficiaries of immune checkpoint inhibitors (ICIs) and provide insight into individualised treatment for COAD.

## INTRODUCTION

Colon adenocarcinoma (COAD) is one of the most widespread malignancies globally, with approximately 1.14 million new cases and 570,000 deaths in 2020 [1]. Current treatment options for COAD include endoscopic resection, surgery, radiotherapy, targeted therapy and immunotherapy [2]. Although early screening and diverse treatment options have significantly improved overall survival in COAD, new cases and deaths from colorectal cancer have been estimated to rise significantly in the next decade [3], adding significantly to the public health challenge. The search for novel biomarkers to improve the clinical outcome of patients with COAD is therefore crucial.

Cancer-associated fibroblasts (CAFs) are key components of the tumour microenvironment (TME) [4, 5], promoting not only the malignant phenotype of cancer but also drug resistance and immune rejection by cancer cells [5, 6]. CAFs play a key role in COAD [7–9], and their consideration as a therapeutic target for cancer has gained widespread attention and recognition [10]. Although satisfactory risk models based on CAFs have been developed to predict prognosis and tumour immune microenvironment (TIME) in individuals with certain cancer types [11–13], they are yet to be implemented for COAD. Therefore, it is significant to construct a satisfactory CAFs-based signature in COAD.

The CAF-related signatures (CAFRs) constructed in the present study are excellent biomarkers for predicting clinical outcomes in individuals with COAD and identifying independent risk factors affecting patient prognosis. Additionally, we explored the biological functions and TIME differences in these CAFRs. Microsatellite instability (MSI) status and tumour mutational burden (TMB) were investigated, and a consensus clustering analysis for CAFRs was performed in patients with COAD. The different clusters effectively differentiated patients’ TIME characteristics, which not only helps to distinguish immune ‘hot’ and ‘cold’ tumours and guides immune checkpoint inhibitors (ICIs) administration but also provides potentially valuable individualised treatment options for patients with cancer.

## MATERIALS AND METHODS

### Data collection

Transcriptome profiling data, simple nucleotide variation data and clinical parameters of individuals with COAD were downloaded from The Cancer Genome Atlas (TCGA) repository (https://portal.gdc.cancer.gov/repository). The downloaded data were collated for follow-up studies using Perl scripts. Transcriptome and corresponding clinical information from the GSE17538 cohort were downloaded from the Gene Expression Omnibus (GEO) (https://www.ncbi.nlm.nih.gov/). Cases in the TCGA and GEO cohorts containing both transcriptomic data and survival data were included in the follow-up study. Immunohistochemical images of CAF-related genes were downloaded from the Human Protein Atlas (HPA, version: 22.0) (https://www.proteinatlas.org) [14]. Specific links to all immunohistochemistry images from the Human Protein Atlas used in this study are provided in Supplementary Table 1. The CAF-related gene set was obtained from The Human Gene Database (https://www.genecards.org/) [13].

### Identification of CAF-related genes in COAD

The mRNA expression matrix of CAF-related genes in the TCGA-COAD cohort was extracted using R (vision 4.2.2), and differentially expressed genes (DEGs) between tumour and normal tissues were further identified (fold change (FC) > 1.5, false discovery rate (FDR) < 0.05). The R package ‘pheatmap’ was utilised to map differential gene volcanoes and mRNA expression heatmaps. Subsequently, the packages ‘limma’, ‘sva’ were utilised to obtain the expression data of the DEGs in the TCGA and GEO cohorts and analyse the intersection of the DEGs expression matrix of the two datasets, respectively. The ‘survival’ and ‘survminer’ packages performed univariate Cox analysis to obtain the prognosis-related CAF-related genes in the TCGA cohort and draw a forest plot (*P* < 0.05), respectively.

### Establishment of CAFRs in COAD

The ‘glmnet’, and ‘survival’ packages were utilised to establish CAFRs in COAD. The optimal prognostic genes in the TCGA cohort were screened using univariate regression and least absolute shrinkage and selection operator (LASSO) algorithms and the resultant genes were utilised to construct CAFRs. The risk score of each sample was obtained through the expression of the CAFRs-related genes and the corresponding regression coefficient. The risk equation used was as follows: $\text{Risk}\;\text{score}=\sum_{i=1}^{n}\text{Coef}(i)\times \text{Expr}(i).$ Coef(*i*) and Expr(*i*) represent the regression coefficient and expression values for each gene in CAFRs, respectively. All individuals were classified into high- and low-risk subgroups based on the median risk score in the TCGA cohort.

### Validation of the CAFRs in COAD

The ‘pheatmap’, ‘survival’ and ‘survminer’ packages were utilised to plot risk heatmaps, risk curves, survival status maps and Kaplan–Meier (K-M) curves for individuals in the TCGA and GEO cohorts. Cox regression evaluated risk scores and clinicopathological parameters to identify independent prognostic variables in the TCGA and GEO cohorts. Additionally, receiver operating characteristic (ROC) curves were drawn utilising the ‘survminer’, ‘survival’ and ‘timeROC’ packages to evaluate the prognostic value of the developed CAFRs based on the size of the area under the curves in the TCGA and GEO cohorts.

### Correlation analysis of the CAFRs with clinical parameters in COAD

To stratify and validate the CAFRs, we further divided patients into two groups based on age, gender and tumour stage. Survival differences between high- and low-risk groups across clinical subgroups were analysed using K-M curves to determine the applicability of the constructed CAFRs to the different subgroups of patients with COAD having different clinical parameters. Finally, the ‘ComplexHeatmap’ was utilised to create a heatmap of the status of different clinical parameters in the two risk subgroups.

### Correlation analysis of the CAFRs and the TIME in COAD

CIBERSORT, an algorithm, implements a machine learning approach for the high-throughput characterisation of different cell types, such as tumour-infiltrating immune cells (TIICs) [15]. The fraction of 22 TIICs was determined using ‘limma’, ‘CIBERSORT’, ‘preprocessCore’, ‘e1071’ and ‘parallel’ and the differences in TIICs between the two subgroups were further analysed. Gene set enrichment analysis (GSEA) enables the enrichment analysis of gene sets with physiological regulatory roles and biological effects [16, 17]. The single sample GSEA (ssGSEA) was performed utilising the ‘GSEABase’ and ‘GSVA’ to estimate immune cell and immune function scores for each sample. Estimation of Stromal and Immune cells in MAlignant Tumor tissues using Expression data (ESTIMATE) analysis is an expression-based tumour purity determination algorithm [18]. Here, the ‘ESTIMATE’ package was utilised to calculate stromal scores and immune scores in the tumour tissue. Subsequently, the ‘ggpubr’ package was employed to draw box plots of stromal, immune and ESTIMATE scores in the risk subgroups.

### Correlation analysis of the CAFRs with MSI and TMB

Genomic hypermutability leads to a molecular tumour phenotype known as MSI [19]. Studies suggest that MSI has the potential as a viable biomarker for ICIs therapy [20]. The ‘ggplot2’, ‘ggpubr’ and ‘plyr’ were utilised to analyse the proportions of microsatellite-stable (MSS), MSI-High and MSI-Low phenotypes in the different risk groups and plot percentage histograms. Additionally, TMB is defined as the total number of somatic mutations per million bases [21] and is used as a biomarker of response to treatment with ICIs in certain solid tumours [22–24]. We analysed TMB levels in the risk groups and plotted box plots.

### GSVA and gene ontology (GO) analysis

The Gene Set Variation Analysis (GSVA) is an algorithm utilised to detect differences in pathway activity among sample populations [25]. GSVA was conducted to obtain the enrichment of Kyoto Encyclopedia of Genes and Genomes (KEGG) pathways in the two risk subgroups, and the correlation between KEGG pathways and signature gene expression was analysed. These analyses were implemented using the R ‘limma’, ‘pheatmap’, ‘GSEABase’, ‘reshape2’, ‘ggplot2’ and ‘GSVA’ packages. Additionally, the DEGs (FC > 2 and FDR < 0.05) between risk groups were determined using ‘limma’. Furthermore, the ‘org. Hs. eg. db’, ‘ggplot2’, ‘enrich’, ‘GOplot’ and ‘clusterProfiler’ were utilised to perform GO and KEGG analysis of DEGs between the risk groups and explore the enrichment of DEGs in cell component, molecular function and biological processes.

### Consensus clustering analysis

The package ‘ConsensusClusterPlus’ was utilised to cluster the COAD samples of the TCGA queue according to the established prognostic characteristics. The packages ‘ggplot2’ and ‘Rtsne’ were utilised for principal component analysis (PCA). The relationship between different COAD clusters and patient survival and TIME was further studied using K-M curves, ESTIMATE, MSI and ssGSEA. Additionally, ‘limma’, ‘reshape2’, ‘ggplot2’ and ‘ggpubr’ were utilised to determine the expression of genes related to immune checkpoints in different clusters, and differential box plots were drawn for immune checkpoints with significant differences (*P* < 0.05).

### Analysis of clinical therapeutic drug sensitivity

The packages ‘pRRophic’ and ‘ggpubr’ were used to obtain the half maximum inhibitory concentration (IC50) of various drugs in the different clusters and draw a differential box chart for the various drugs (*P* < 0.001). They were also used to explore the potential clinical significance of cluster analysis based on the CAFRs in drug treatment.

### COAD tissue samples

Colon tumor tissues and adjacent normal tissues were acquired from the First Affiliated Hospital of Anhui Medical University (Hefei, China). All colon tumors were histologically confirmed as COAD. The study was approved by the Medical Ethics Committee of the First Affiliated Hospital of Anhui Medical University (No. PJ20230861). All enrolled COAD patients provided written informed consent.

### Quantitative real-time PCR

RT-qPCR was used to measure the mRNA level in tumor tissues and adjacent normal tissues. RNA extraction and RT-qPCR was performed as previously described [26]. Briefly, the total RNA was isolated using RNA isolation reagent (Takara Bio, Japan), and reverse transcribed into cDNA with PrimeScript^™^ RT Master Mix (Takara Bio, Japan) following the manufacturer’s protocol. Quantitative real-time PCR (qPCR) was performed using SYBR-Green qPCR Master Mix (Vazyme Bio, China). The primer sequences for the CAFRs-related genes used in the experiments were listed in Supplementary Table 2. The GAPDH was used as an internal control for normalization. Relative gene expression was estimated according to the 2^−ΔΔCt^ method.

### Statistical analysis

All statistical analyses were performed using R software (version 4.1.2) and the corresponding R packages. K-M method was utilised to plot the survival curves of different subgroups. The correlation between different continuous variables was assessed by Pearson correlation test. The Wilcoxon test was utilised for comparing two groups. *P* < 0.05 was considered as statistically significant for a difference.

### Data availability statement

All data presented in this study are available from the corresponding author upon reasonable request.

## RESULTS

### Identification of CAF-related genes in COAD

**Figure 1:**
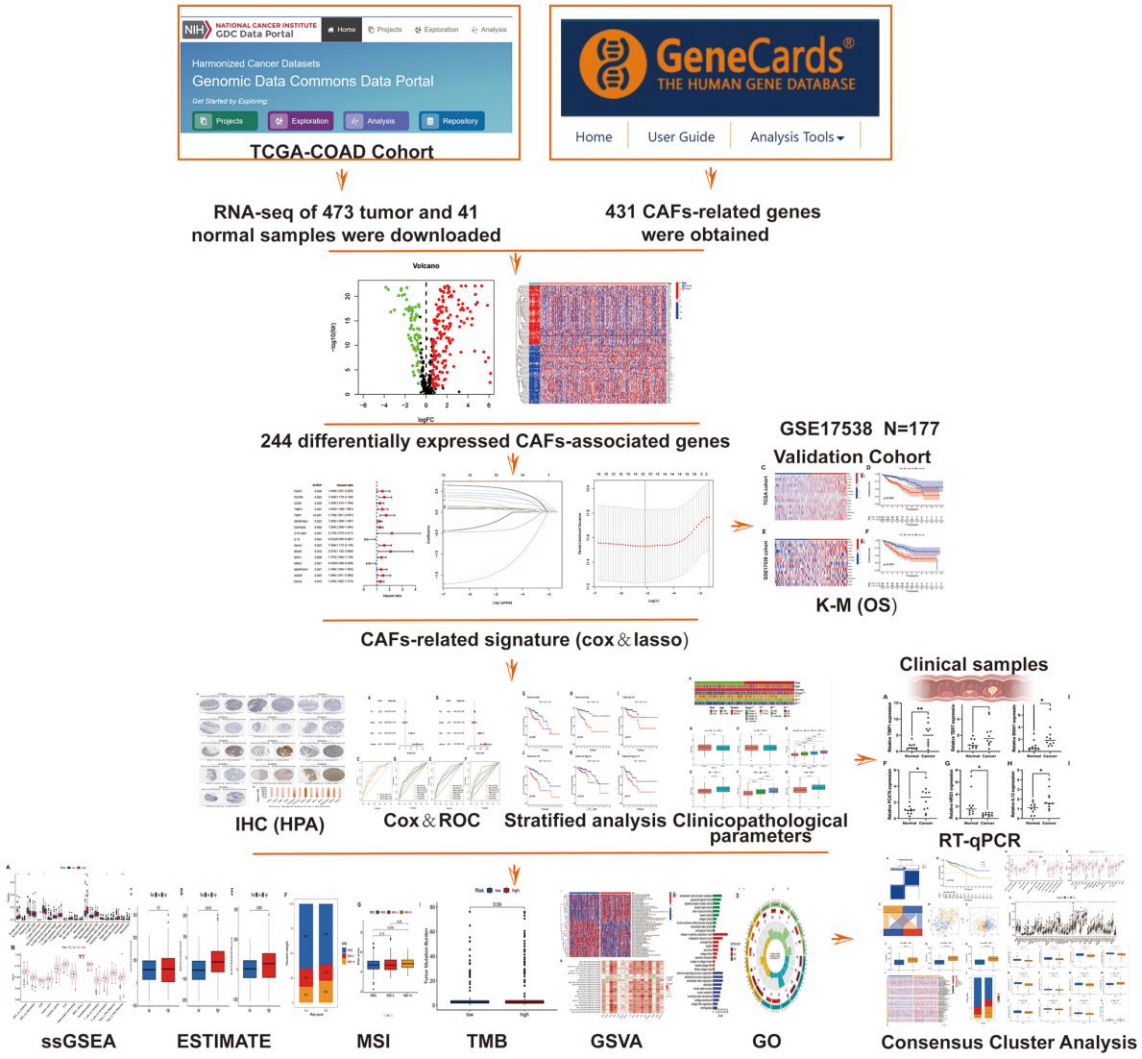
Flow chart.

**Figure 2:**
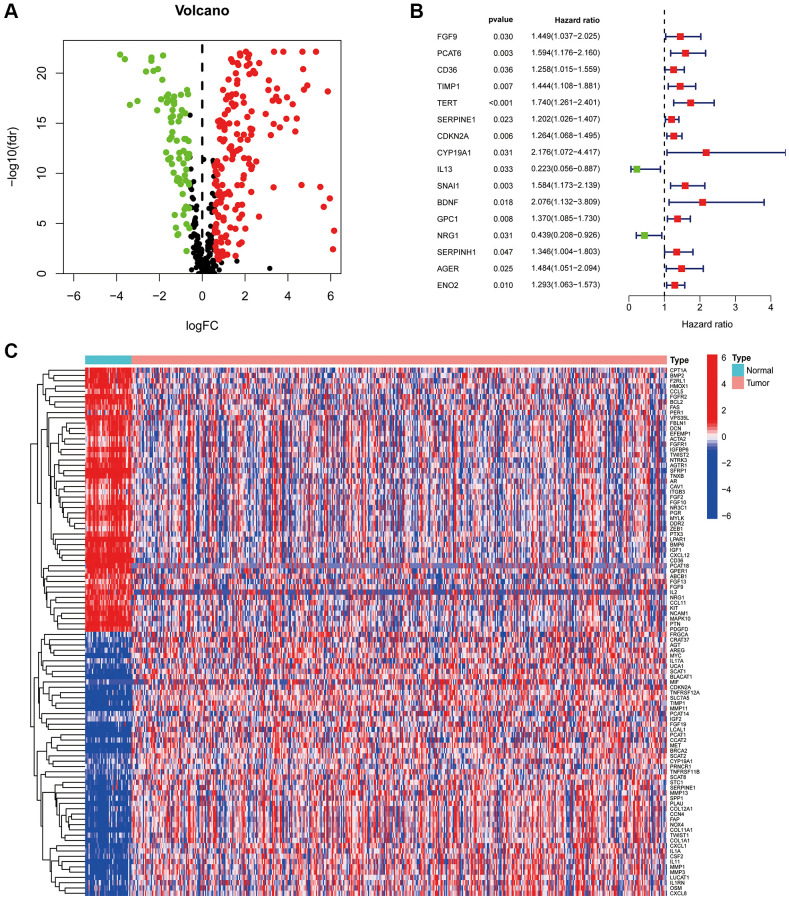
**CAF-related genes in COAD.** (**A**) The volcano plot of 244 CAFs-associated genes shows differential expression. (**B**) The risk forest plot showed that 16 CAF-related genes were associated with COAD prognosis (**C**) Heat map of differentially expressed CAF-related genes.

The study flow chart is illustrated in Figure 1. A total of 473 COAD tumour samples and 41 normal samples were acquired from the TCGA database with relevant data. Overall, 431 CAF-related genes were acquired from the Genecards, all with relevance scores greater than 5 (Appendix 1). A total of 244 CAF-related genes were differentially expressed in COAD tumours and normal tissues, of which 172 were upregulated and the remaining downregulated (Figure 2A). The Cox regression indicated that 16 CAF-related genes were associated with the overall survival (OS) of COAD (Figure 2B). The expression patterns of the 50 CAF-related genes with the highest up- and down-regulation folds among the DEGs are presented as a heat map (Figure 2C).

### Construction of the CAFRs in COAD

**Figure 3:**
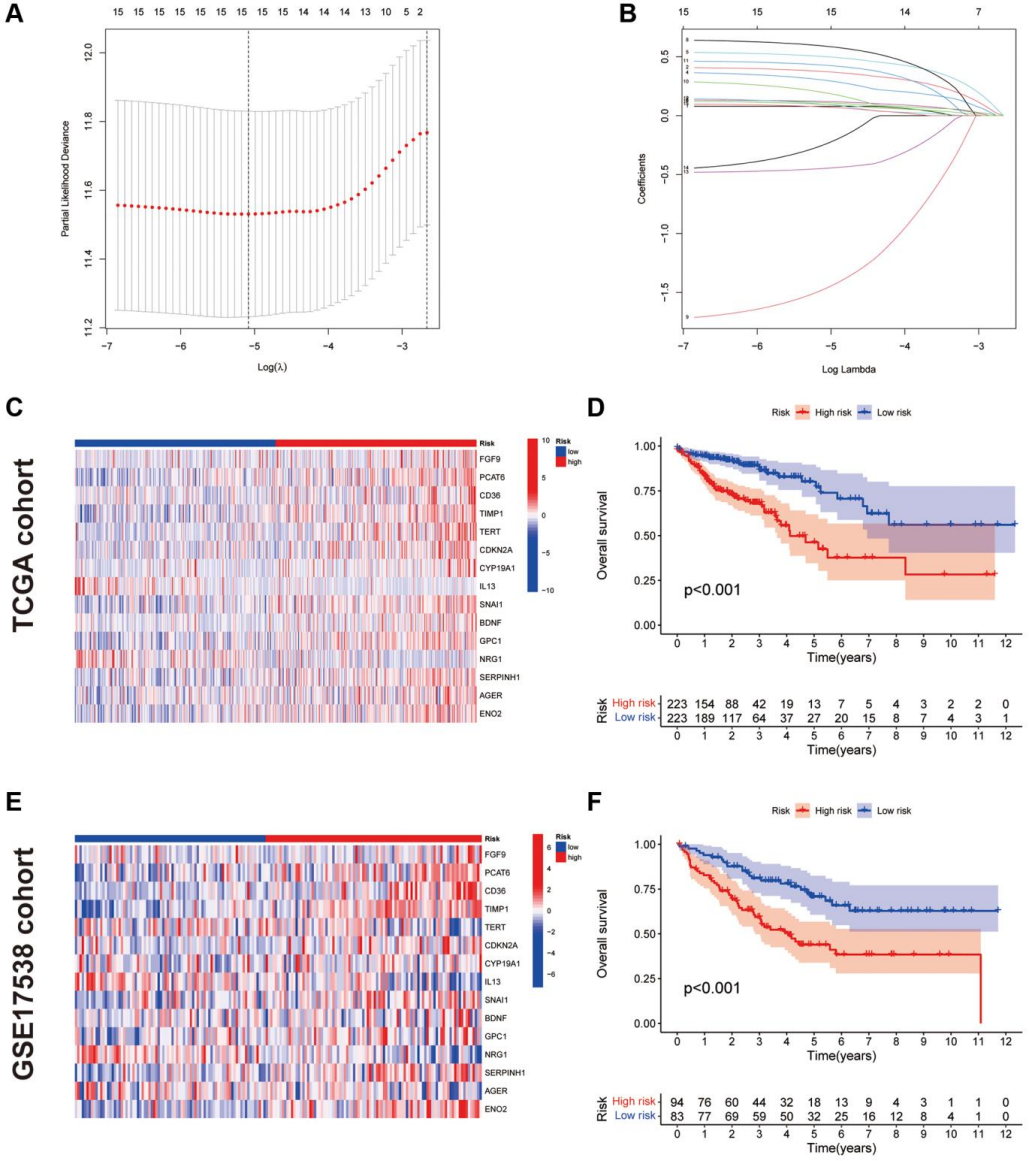
**Construction and validation of CAFRs.** (**A**, **B**) The coefficient and partial likelihood deviance of the prognostic signature. (**C**) Heat map of the expression of the 15 CAFs-associated genes in the TCGA cohort. (**D**) K–M curve for OS in the TCGA cohort. (**E**) Heat map of the expression of the 15 CAFs-associated genes in the GEO cohort. (**F**) K–M curve for OS in the GEO cohort.

To avoid overfitting, the LASSO algorithm was utilised (Figure 3A, 3B), identifying 15 CAF-related genes for CAFRs construction (Table 1). The K-M curves of 15 signature-related genes in the TCGA-COAD cohort further confirmed the relationship between the expression of these genes and the survival of patients with COAD (Supplementary Figure 1). Additionally, box line plots demonstrate the differential expression status of CAFRs-related genes in COAD tumor tissues and normal tissues (Supplementary Figure 2).

**Table 1:** LASSO regression coefficients for CAFRs.

**CAFs-related genes**	**Coefficient**
FGF9	0.0787497
PCAT6	0.3741071
CD36	0.1114249
TIMP1	0.3006318
TERT	0.5012208
CDKN2A	0.1204453
CYP19A1	0.5903825
IL13	−1.4691038
SNAI1	0.1950326
BDNF	0.4283392
GPC1	0.0962728
NRG1	−0.4469785
SERPINH1	−0.2432318
AGER	0.0734801
ENO2	0.0958250

The risk score for each case was obtained by calculating the risk regression coefficient and expression of each gene in the CAFRs. Risk score = FGF9 × (0.0787497) + PCAT6 × (0.3741071) + CD36 × (0.1114249) + TIMP1 × (0.3006318) + TERT × (0.5012208) + CDKN2A × (0.1204453) + CYP19A1 × (0.5903825) + IL13 × (−1.4691038) + SNAI1 × (0.1950326) + BDNF × (0.4283392) + GPC1 × (0.0962728) + NRG1 × (−0.4469785) + SERPINH1 × (-0.2432318) + AGER × (0.0734801) + ENO2 × (0.0958250).

**Figure 4:**
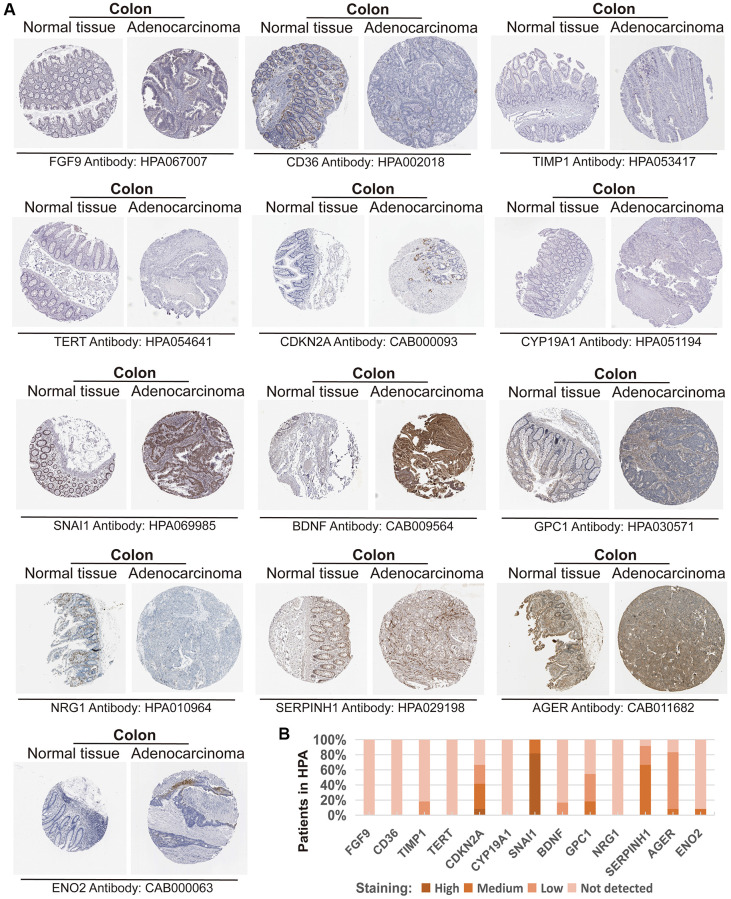
**Expression of the protein encoded by the CAFRs-related gene in the Human Protein Atlas (HPA).** (**A**) Immunohistochemical images of the protein encoded by some CAFRs-related genes in COAD normal and tumour tissue in the HPA. (**B**) The proportion of the protein encoded by some CAFRs-related genes that is expressed in the COAD of the HPA.

We further validated the CAFRs in the TCGA and GSE17538 cohorts. The expression status of the 15 signature genes in the two cohorts is shown in heat maps (Figure 3C, 3E). K-M analyses of the two cohorts revealed a significantly poorer clinical outcome for individuals with COAD in the high-risk subgroup (Figure 3D, 3F). In addition, immunohistochemical images of the HPA database showed the expression of proteins encoded by some of the signature-related genes in COAD normal and tumor tissues (Figure 4A, 4B).

### Assessment of the CAFRs in COAD

**Figure 5:**
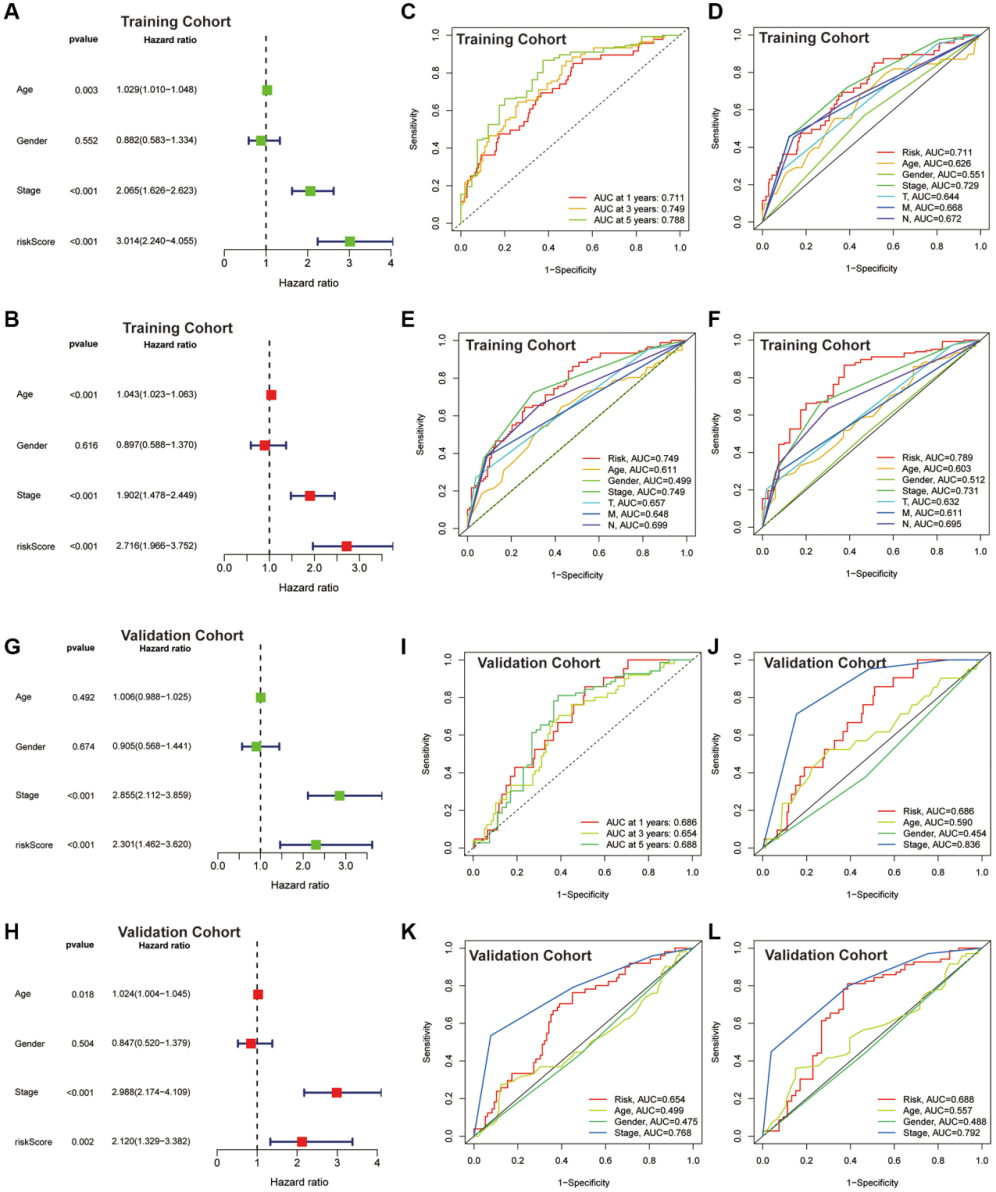
**Assessment of the CAFRs.** (**A**, **B**) Forest plot for univariate and multivariate Cox regression analyses in the TCGA-COAD cohort. (**C**) ROC curves of 1-, 3- and 5-year survival for the CAFRs in the TCGA-COAD cohort. (**D**–**F**) Comparison of the prediction accuracy of the CAFRs with age, gender, TNM-stage, T-stage, N-stage and M-stage at 1-, 3- and 5- years in the TCGA-COAD cohort. (**G**, **H**) Forest plot for univariate and multivariate Cox regression analyses in the GEO cohort. (**I**) ROC curves of 1-, 3- and 5-year survival for the CAFRs in the GEO cohort. (**J**–**L**) Comparison of the prediction accuracy of the CAFRs with age, gender and stage in the GEO cohort.

Risk scores based on the CAFRs were identified as an independent prognostic indicator for the TCGA-COAD cohort using univariate and multivariate Cox regression, with hazard ratio values of 3.014 (2.240–4.055; *P* < 0.001) and 2.716 (1.966–3.752; *P* < 0.001) (Figure 5A, 5B). The tumour stage was also an independent factor (*P* < 0.001). The ROC curves were utilised to evaluate the specificity and sensitivity of the CAFRs for COAD prognosis. The area under the curve values for the CAFRs predicting OS at 1-, 3- and 5- years were 0.711, 0.749 and 0.788 (Figure 5C–5F). Additionally, Cox regression analysis and ROC curves of the GEO validation cohort further validated that CAFRs is an independent prognostic factor for COAD with good prognostic predictive efficacy (Figure 5G–5L).

### Correlation of the CAFRs with clinical parameters in COAD

**Figure 6:**
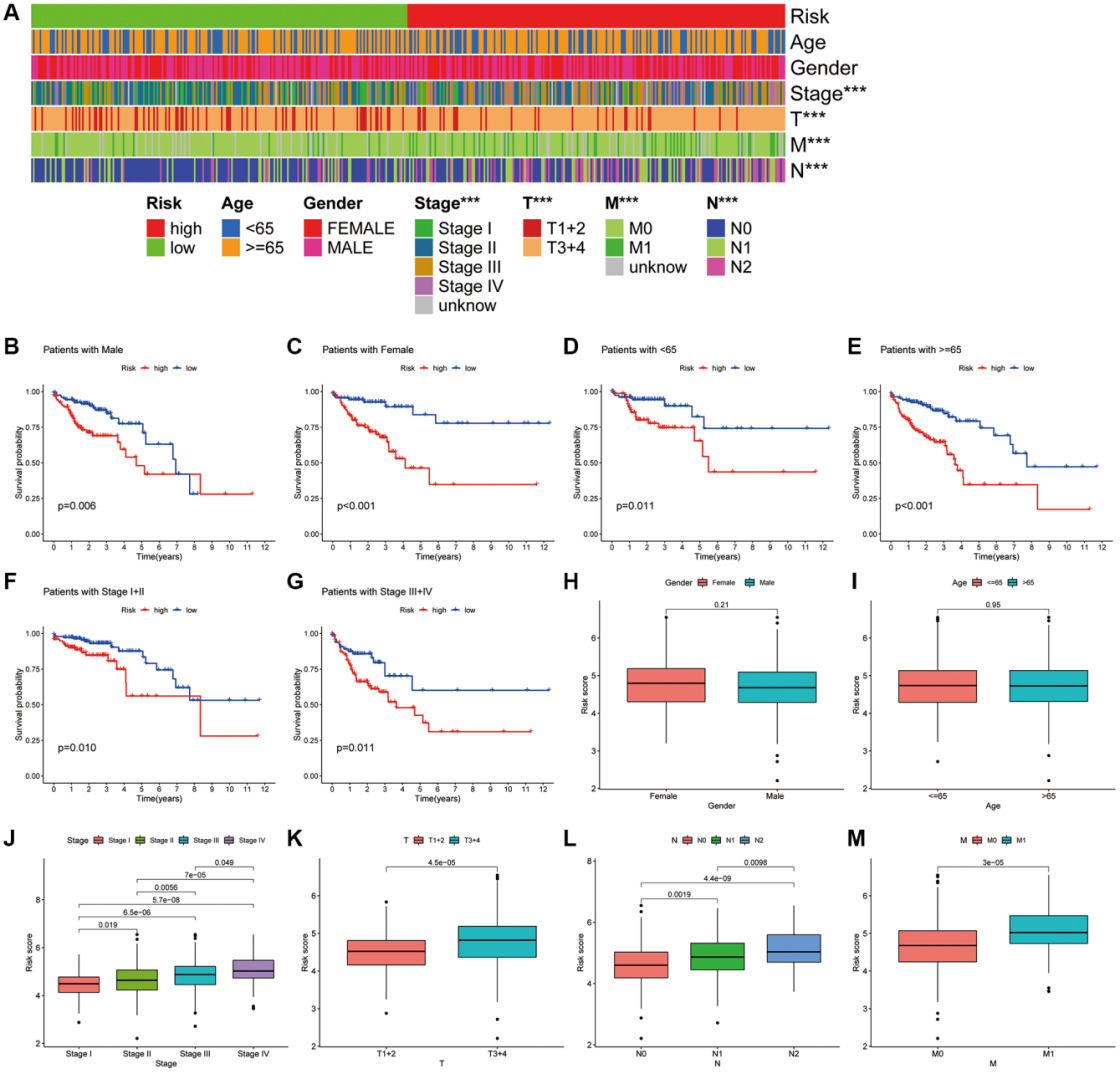
**Association of CAFRs with clinicopathological parameters in COAD.** (**A**) A strip chart of the associations between risk status and clinical parameters. (**B**–**G**) K–M curves of low- and high-risk subgroups sorted by gender, age and TNM stage. (**H**–**M**) Box plot of the difference in risk scores by gender, age, TNM-stage, T-stage, N-stage and M stage.

We further analysed the correlation of the CAFRs with the clinical parameters. The heat map shows the status of different clinical factors in the risk subgroups (Figure 6A). To further analyse whether the CAFRs apply to individuals with different clinicopathological factors, survival analyses were performed on different clinical subgroups of patients. Stratified K-M curves indicated that individuals with different gender, age and tumour stages had worse prognoses in the high-risk group (Figure 6B–6G), demonstrating the stability of the CAFRs. Additionally, box plots of risk scores for different clinical subgroups showed that as the tumour stage increased (stages I-IV), the risk score also increased significantly. Moreover, the same results were observed for the T-, N- and M-stage. However, the risk scores did not differ significantly in the age and gender subgroups (Figure 6H–6M).

### Correlation of the CAFRs with TIME in COAD

**Figure 7:**
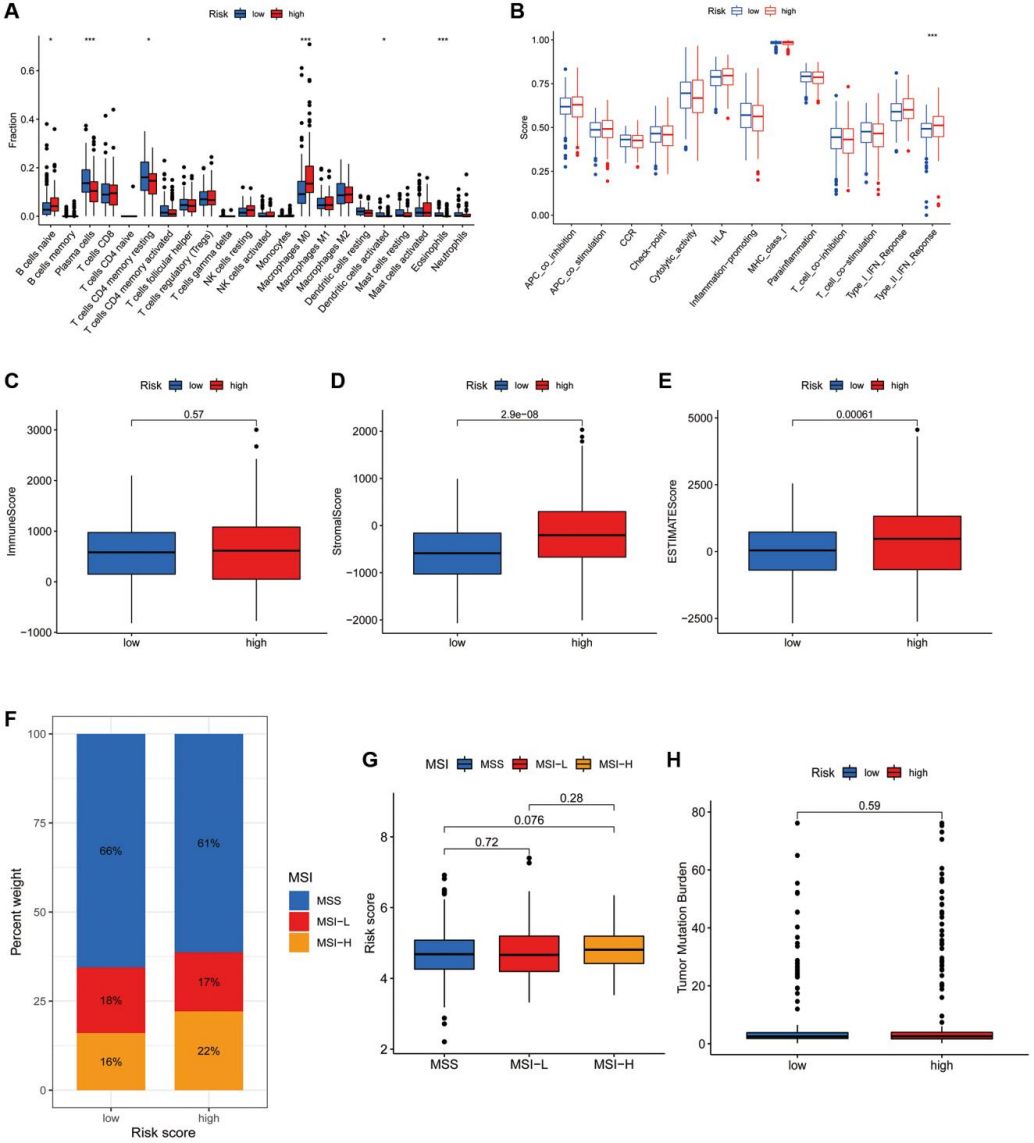
**Association of the CRFRs with the immune microenvironment of COAD.** (**A**) Box plot showing differences in immune cells between the high- and low-risk subgroups using the CIBERSORT algorithm. (**B**) Box plot showing differences in immune-related functions between the high- and low-risk groups using the ssGSEA algorithm. (**C**–**E**) Stromal score, immunity score and ESTIMATE score in the two risk subgroups. (**F**) Histogram of proportions showing the proportion of patients with MSS, MSI-L and MSI-H in the high- and low-risk subgroups. (**G**) Box plot of differences in risk scores for patients in the MSS, MSI-L and MSI-H subgroups. (**H**) Box plot of TMB difference for the high- and low-risk subgroups. ^*^P < 0.05, ^**^P < 0.01 and ^***^P < 0.001.

CIBERSORT algorithm revealed that naive B cells, plasma cells, resting CD4+ T cells, M0 macrophages, activated dendritic cells and eosinophils differed significantly between the high- and low-risk subgroups (Figure 7A). However, most of the other immune infiltrating cells did not differ significantly in the risk groups. ssGSEA also showed no significant difference in most immune-related functions between the high- and low-risk groups, with the exception of type II IFN response (Figure 7B). ESTIMATE analysis revealed that the stromal and ESTIMATE scores were significantly higher in the high-risk group but the immune score did not differ between the two subgroups (Figure 7C–7E).

### Correlation of the CAFRs with MSI and TMB in COAD

MSI status closely correlates with immunotherapy response in gastroenterology tumours. The histogram of proportions shows that the proportions of MSS, MSI-L and MSI-H in the low-risk subgroup were 66%, 18% and 16%, respectively, while the values were 61%, 17% and 22%, respectively, in the high-risk subgroup (Figure 7F). Furthermore, there was no significant difference in the risk scores of individuals in the MSS, MSI-L and MSI-H groups (Figure 7G). Additionally, there was also no significant difference in TMB status between the risk groups (Figure 7H).

### GSVA and GO analysis of the CAFRs in COAD

**Figure 8:**
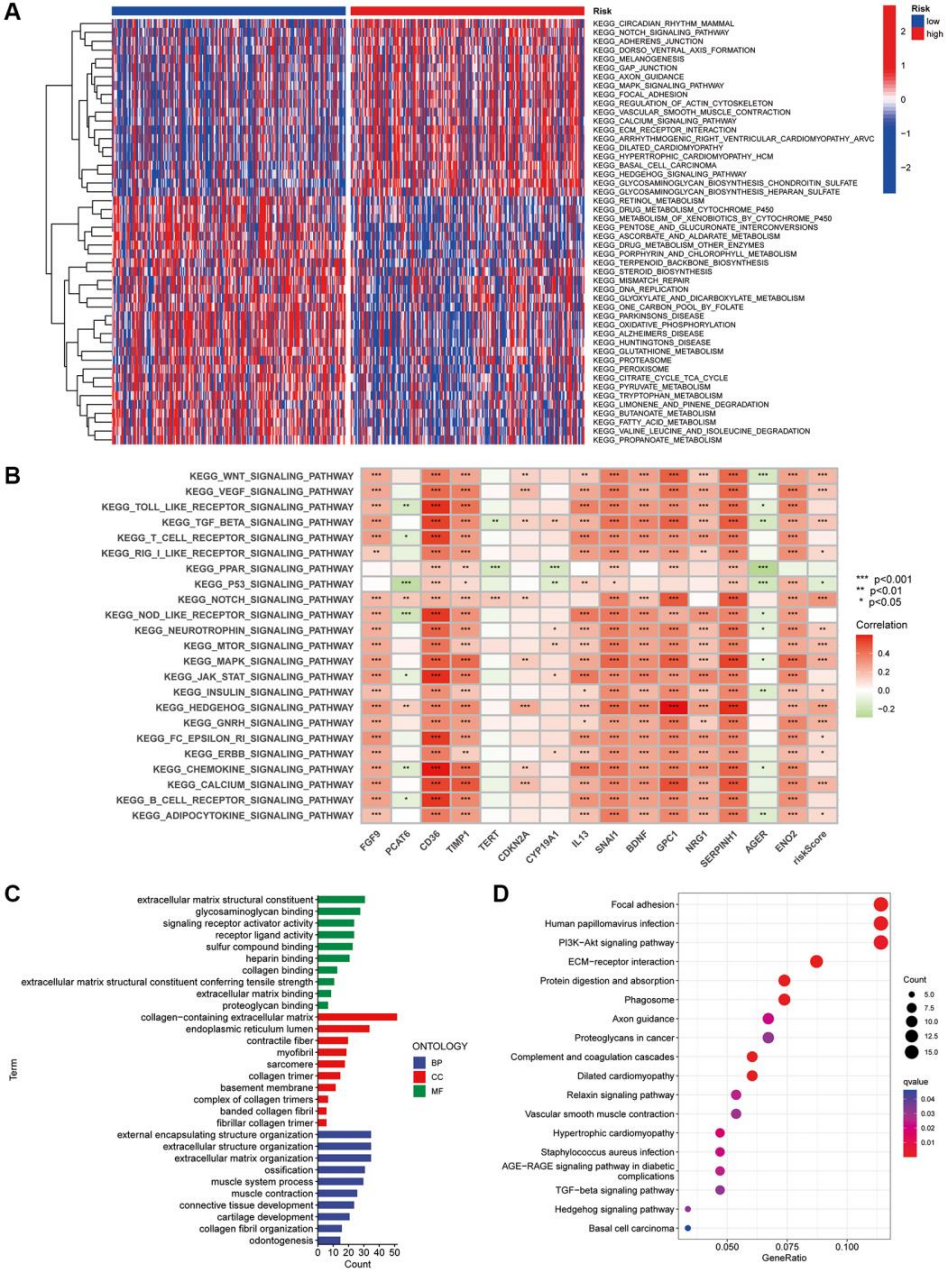
**GSVA and GO analysis.** (**A**) Heat map of functional pathway enrichment differences between the two risk groups. (**B**) Heat map of the correlation between the expression of signature genes and signalling pathways. (**C**) GO analysis shows the enrichment of DEGs between the high- and low-risk subgroups. (**D**) KEGG analysis shows the enrichment of DEGs between the two risk subgroups.

GSVA investigated the biological differences between the risk groups and revealed that the high-risk subgroup was enriched in pathways such as circadian rhythm, Notch signalling pathway, MAPK signalling pathway, actin cytoskeleton regulation, calcium signalling pathway, extracellular matrix receptor interactions, basal cell carcinoma and Hedgehog signalling pathway, which are associated with tumour malignancies. Additionally, the metabolism of retinol; toxic metabolism of cytochrome P450; interconversion of pentose and glucuronide, ascorbic acid and aldehyde; metabolism of drugs; metabolism of glutathione; metabolism of fatty acids; mismatch repair and DNA replication were enriched in the low-risk subgroup (Figure 8A). Furthermore, spearman correlation analysis showed a strong correlation between the expression of the 15 genes in the CAFRs and signalling pathways related to tumour evolution (Figure 8B).

Additionally, we investigated the biological functions of DEGs in the different risk groups. In terms of biological processes, the DEGs were enriched in extracellular matrix structural constituent, signalling receptor activator activity, glycosaminoglycan binding, receptor-ligand activity, sulfur compound binding and extracellular matrix binding. Regarding molecular function, the DEGs were enriched in the external encapsulating structure organisation, extracellular structure organisation, extracellular matrix organisation, ossification, connective tissue development and other functions. Furthermore, the DEGs were enriched in cellular components such as the endoplasmic reticulum lumen, contractile fibre and myofibril (Figure 8C). Finally, KEGG analysis revealed that DEGs were enriched in pathways including focal adhesion, PI3K-Akt signaling pathway, ECM-receptor interactions, and protein digestion and absorption (Figure 8D).

### Consensus clustering based on the CAFRs

**Figure 9:**
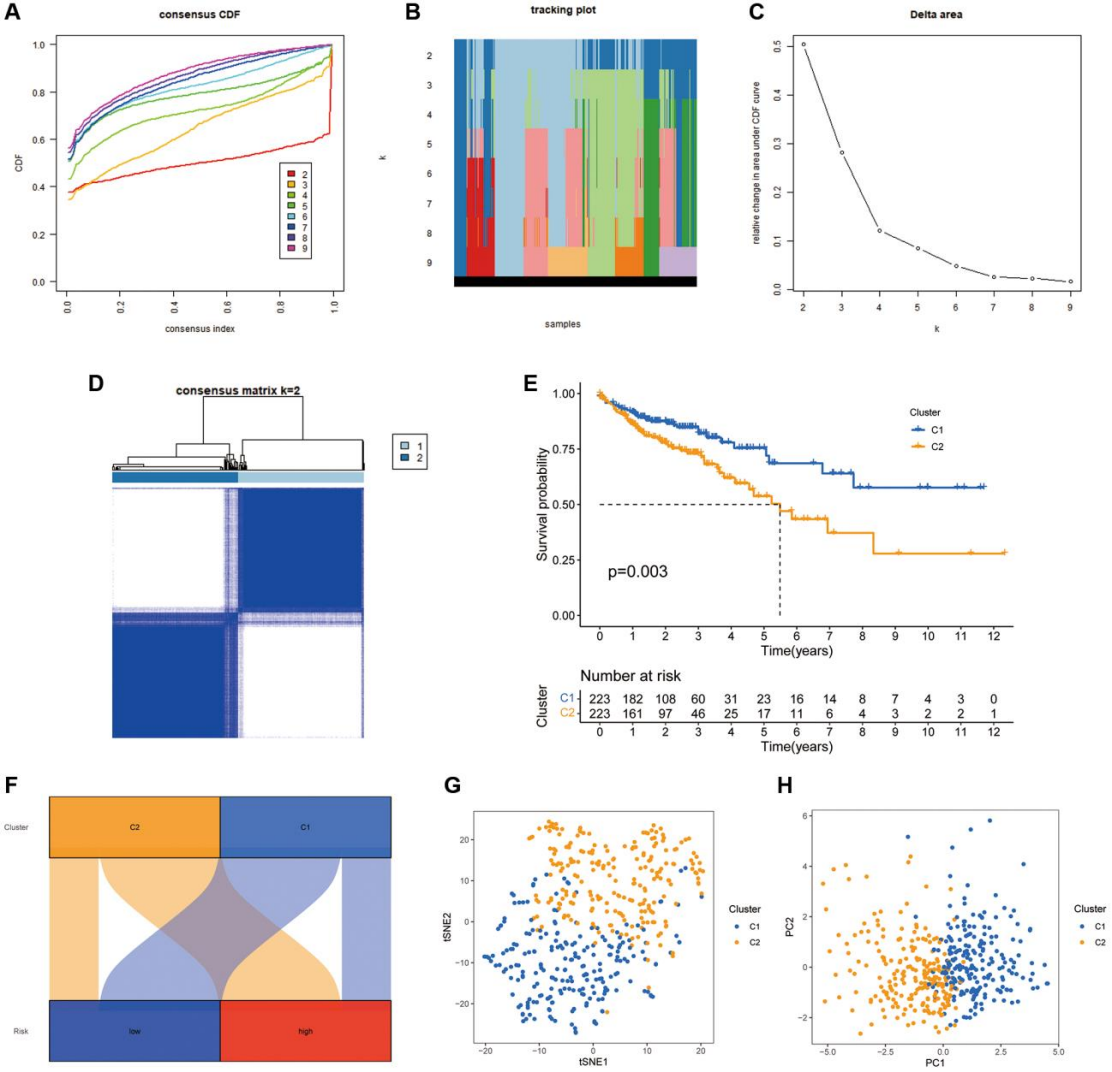
**COAD classification based on the CRFRs.** (**A**) The cumulative distribution function curves for k = 2–9. (**B**) The tracking plot of consistent clustering. (**C**) The elbow plot showing relative change in area under the cumulative distribution function curve. (**D**) Consensus clustering matrix for k = 2. (**E**) K–M curves of the two clusters. (**F**) Sankey diagram of the association between the risk groups and clusters. (**G**, **H**) PCA and tSNE analyses of the clusters.

There is growing evidence that tumour subgroups derived from consensus clustering analysis have different TIME landscapes and influence the response to tumour immunotherapy [27, 28]. All patients in the TCGA-COAD cohort were divided into k (k = 2–9) clusters using ConsensusClusterPlus. According to the cumulative distribution function curve of the consensus scores, the best classification occurs when k = 2. Therefore, all patients in the TCGA-COAD cohort were classified into cluster 1 (*n* = 223) and cluster 2 (*n* = 223), when the variability was lowest within clusters and highest between clusters (Figure 9A–9D). The K-M curves revealed that individuals in cluster 2 have worse survival rates than those in cluster 1 (*P* = 0.003) (Figure 9E). The Sankey plots revealed that the majority of individuals in cluster 1 were in the low-risk group, while the majority of patients in cluster 2 were in the high-risk group (Figure 9F). The findings indicate that the cluster typing developed can help determine the prognosis of patients with COAD. Additionally, the PCA and tSNE significantly distinguished the distributional features of the two clusters (Figure 9G, 9H).

**Figure 10:**
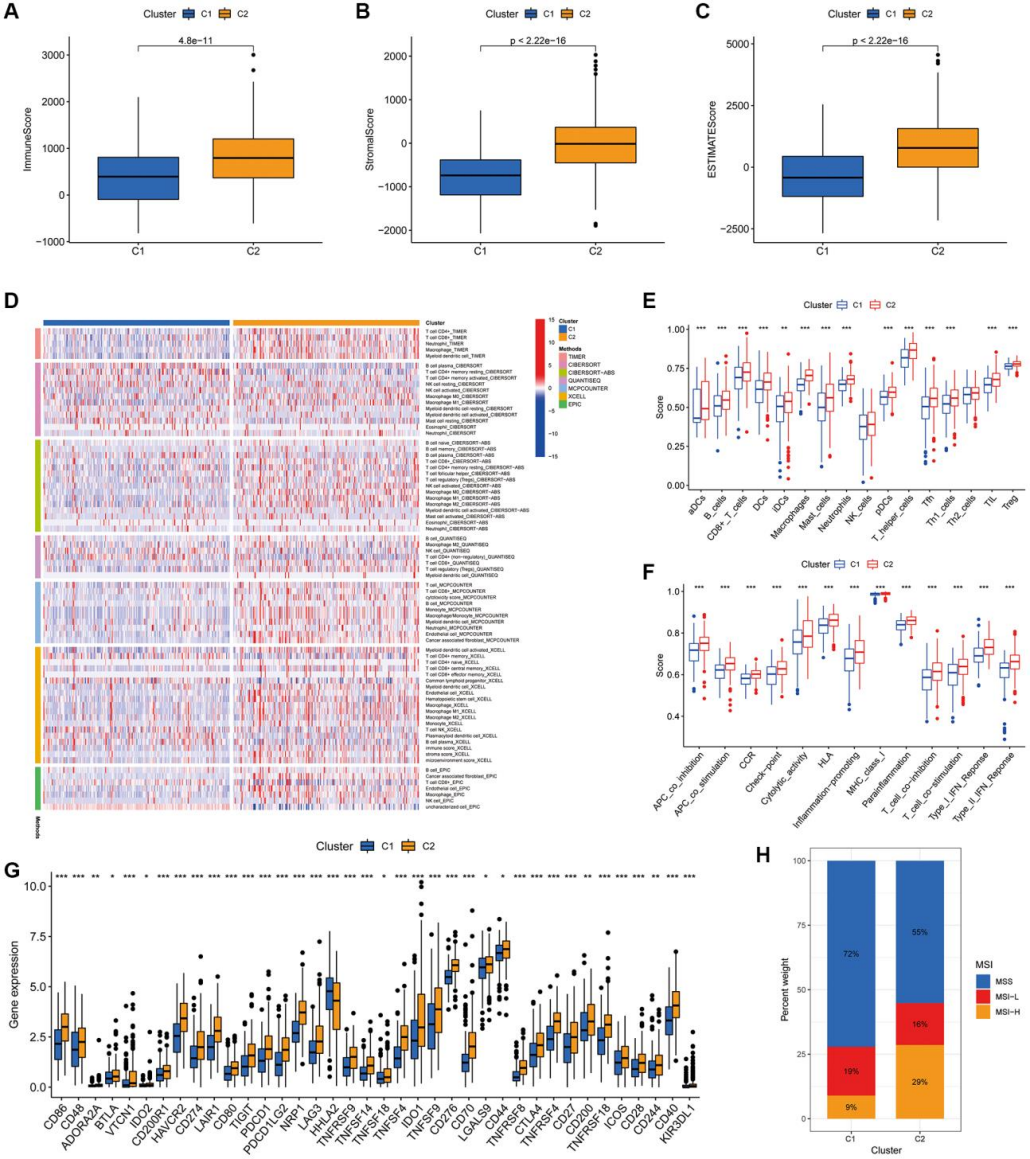
**Association of the two clusters with the TIME.** (**A**–**C**) Immune, stromal and ESTIMATE scores in the two clusters. (**D**) Heat map of the proportion of different types of immune cells. (**E**) Box plot showing differences in immune cells between the two clusters using the ssGSEA algorithm. (**F**) Box plot showing differences in immune-related functions between the two clusters using the ssGSEA algorithm. (**G**) Expression of immune checkpoint markers in the two clusters. (**H**) Histogram of proportions showing the proportion of patients with MSS, MSI-L and MSI-H in the two clusters. ^*^P < 0.05, ^**^P < 0.01 and ^***^P < 0.001.

We further explored the impact of cluster analysis on the TIME of COAD tumours using ESTIMATE analysis, which revealed that the immune, stromal and ESTIMATE scores were significantly higher in cluster 2 (Figure 10A–10C). The heat map showed that the majority of immune infiltrating cells were significantly less abundant in cluster 1 than in cluster 2 (Figure 10D). Additionally, ssGSEA validated these findings, suggesting that both immune-related functions and immune cell infiltration were significantly stronger in cluster 2 (Figure 10E, 10F). Furthermore, most immune checkpoints were significantly more highly expressed in cluster 2 (Figure 10G). This suggests that patients in cluster 2 were more likely to benefit from ICIs compared to the cluster 1 population. Furthermore, the histogram of proportions revealed that the proportions of MSS, MSI-L and MSI-H in cluster 1 were 72%, 19% and 9%, respectively, while the values were 55%, 16% and 29%, respectively, in cluster 2 (Figure 10H).

**Figure 11:**
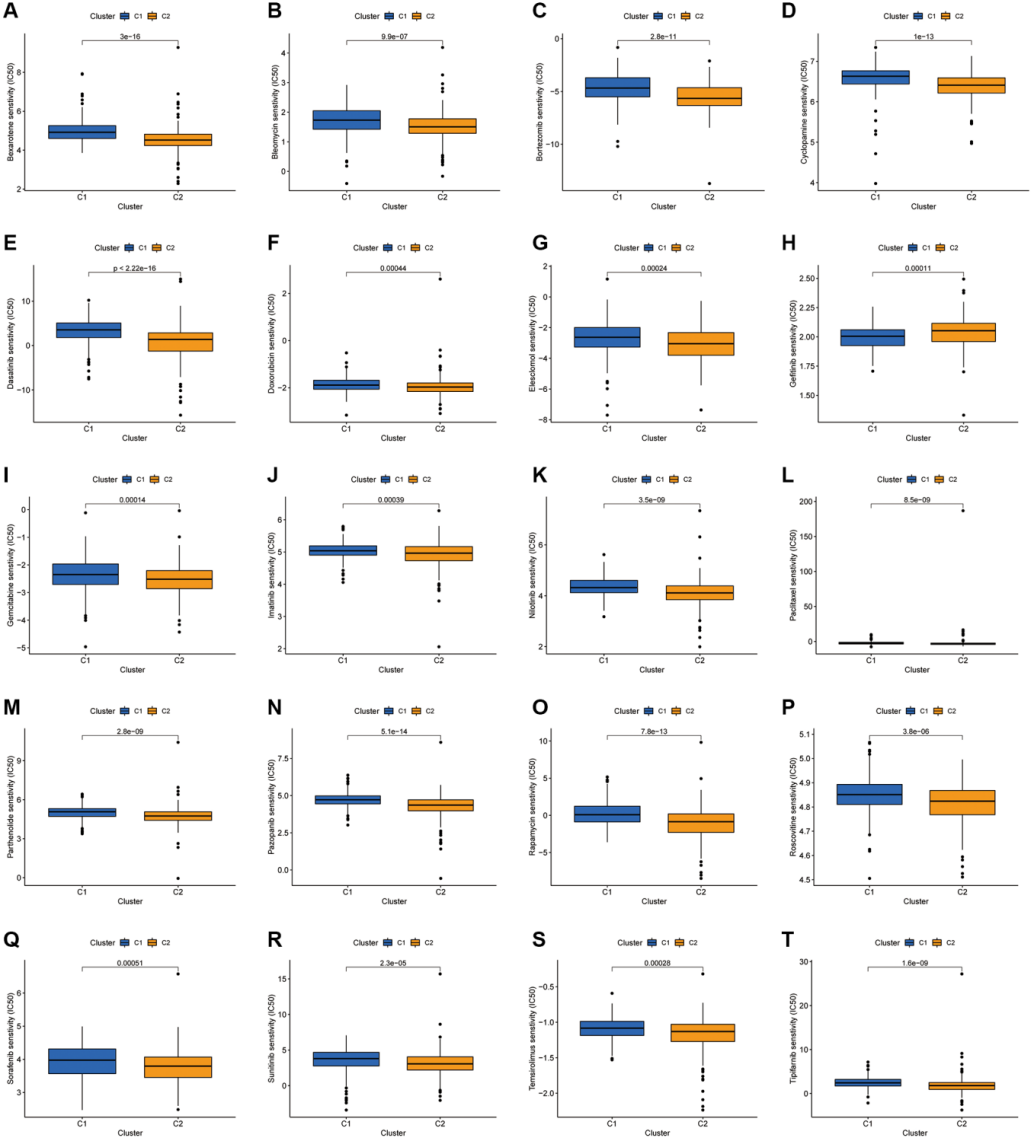
**Investigation of drug sensitivity.** (**A**–**T**) Boxplots of IC50 values for different agents in the two clusters.

Drug sensitivity analysis of the two clusters revealed variations in IC50 for numerous chemical and targeted anti-cancer agents between the clusters (*P* < 0.001) (Figure 11A–11T). These findings imply that our clustering analysis could offer a basis for the selection of targeted therapeutic regimens and chemotherapeutic agents for patients with COAD.

### Validation of CAFRs genes expression levels in COAD tissues

**Figure 12:**
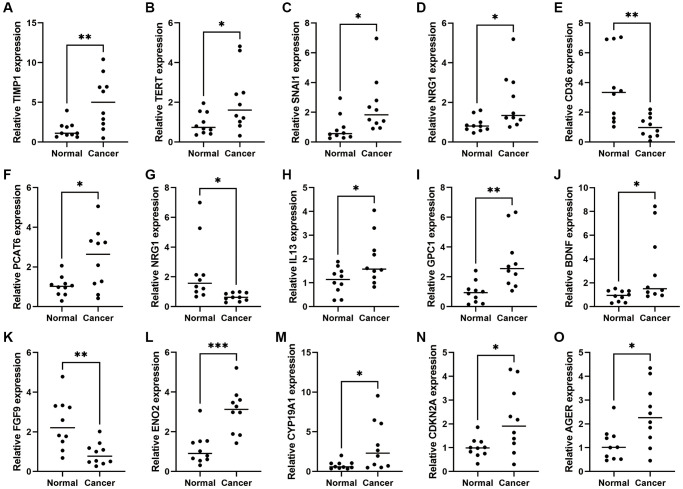
**Expression of CAFRs-related genes in COAD and adjacent normal tissues.** (**A**–**O**) mRNA expression levels of the 15 CAFRs-related genes in COAD and adjacent normal tissues (*n* = 10). ^*^P < 0.05, ^**^P < 0.01 and ^***^P < 0.001.

To further investigate the expression levels of CAFRs genes in COAD clinical tissues, we examined the mRNA expression levels of CAFRs genes in COAD tumor tissues and adjacent normal tissues. The qRT-PCR results showed that the mRNAs of all CAFRs genes were differentially expressed in COAD tumor tissues and adjacent normal tissues, among which CD36, NRG1 and FGF9 were highly expressed in adjacent normal tissues, whereas TIMP1, TERT, CDKN2A, PCAT6, CYP19A1, IL13, SNAI1, BDNF, GPC1, SERPINH1, AGER, and ENO2 were highly expressed in the tumor tissues (Figure 12A–12O).

## DISCUSSION

Malignant tumours remain one of the major diseases that pose a serious threat to human health. The evolution of tumours is determined by a combination of the intrinsic properties of the tumour cells and the external environment consisting of various other components in the TME [29]. CAFs are a major component of the TME, interacting extensively with tumour cells and influencing other components of the TME [30]. CAFs not only play a role in promoting tumour proliferation, metastasis and invasion but also in inducing anti-tumour drug resistance and immunosuppression [31, 32]. Additionally, growing evidence suggests that CAFs are strongly associated with the efficacy of tumour immunotherapy [13, 33, 34]. Zheng et al. reported that CAFs correlated with CD8+ T cells in the TME and also that the CD8+ T cell/CAFs ratio influenced the response to immunotherapy [35]. Furthermore, targeted therapy against CAFs is considered an effective strategy to improve the efficacy of immunotherapy [36]. Therefore, it is vital to understand the role of CAFs in assessing the prognosis and immunotherapy efficacy of patients with tumours.

In this study, we constructed CAFRs to predict the prognosis of patients with COAD. Patients with COAD were categorised into high- and low-risk subgroups based on risk scores in the CAFRs. Additionally, we validated the prognostic predictive value of CAFRs in COAD cohorts and further assessed the signature using a range of methods, including univariate and multivariate Cox regression analysis and time-dependent ROC curves. The findings suggest that the constructed CAFRs have reliable and excellent prognostic predictive power for COAD.

Immune checkpoints can send ‘off’ signals to suppress immune function, thereby allowing tumour cells to evade immune killing [37, 38]. Recently, ICIs have been increasingly used in the treatment of patients with gastrointestinal tumours, displaying promising efficacy. In particular, ICIs represented by the anti-PD1/PD-L1 pathway have been most effective in patients with mismatch repair deficiency (dMMR)/MSI-H and have been approved for the second-line treatment of metastatic COAD with dMMR/MSI-H [39]. However, the majority of patients represented by pMMR/MSS not only responded poorly to ICIs but also caused disease progression in some patients. It is therefore important to explore biomarkers for predicting and evaluating the response to treatment with ICIs in COAD, thereby benefitting personalised treatment regimens.

Previous studies have confirmed that the molecular subtype of the tumour correlates with the clinical outcome and immune microenvironment characteristics of patients [40, 41]. To analyse the differences in survival and immune landscape of patients with different subtypes of COAD, we performed consensus clustering analysis based on the constructed CAFRs and divided the patients into two clusters. Further analysis revealed that most immune effector cells were more infiltrated in cluster 2 compared to cluster 1. Additionally, our study shows that most immune checkpoints were significantly highly expressed in cluster 2, suggesting that cluster 2 has a highly immunosuppressive microenvironment that promotes the immune escape of tumour cells, which is also corroborated with the poor clinical outcomes of this population.

Despite that the promising effects of ICIs, their low overall efficiency is an urgent issue for clinical immunotherapy. The sparse infiltration of effector immune cells in tumour tissues, known as ‘immune cold tumours’, is considered to be the main factor for the low efficiency of ICIs [42]. Contrastingly, ‘immune hot tumours’ are characterised by a high infiltration of effector immune cells and the activation of immune checkpoints, and respond better to ICIs [43]. Taken together, patients in cluster 2 were more consistent with the characteristics of an ‘immune hot tumour’. Furthermore, in terms of MSI, cluster 2 had up to 29% of patients with MSI-H, which was higher than cluster 1 (9%). This further validates that cluster 2 could be a beneficial population for treatment with ICIs. Thus, our cluster analysis not only facilitates the prediction of prognosis and immune microenvironmental characteristics of different subtypes of COAD but also provides a basis for the identification of a population that is advantageous for the treatment of ICIs.

Patients with advanced COAD usually choose chemotherapy to control the progression of their disease; however, in some patients, efficacy is reduced after conventional first- and second-line standard chemotherapy. With rapid advances in drug development, molecularly targeted drugs with different mechanisms of action have been developed and are commonly used to treat patients with advanced COAD who have failed second-line therapy. Meanwhile, patients who show progress after standard treatment are often treated clinically with a combination of drugs with different mechanisms of action. Therefore, the rational arrangement of different drugs in combination with personalised treatment regimens is crucial for patients with advanced COAD. Notably, most of the targeted drugs, including Dasatinib, Imatinib, Nilotinib, Pazopanib, Sorafenib, Sunitinib and Tipifarnib, had lower IC50s in cluster 2, suggesting that cluster 2 could also be a beneficiary population for small molecule tyrosine kinase inhibitors.

## CONCLUSION

The CAFRs and CAFRs-based clusters established can effectively predict the prognosis of patients with COAD and differentiate TIME characteristics in patients. This aids in distinguishing immune ‘hot’ and ‘cold’ tumours and guides ICI administration. Furthermore, the constructed signature provides valuable individualised treatment options for patients with cancer. Nevertheless, the therapeutic potential of CAFRs in clinical settings requires further validation in the future using prospective clinical trials with large samples.

## Supplementary Materials

Supplementary Figures

Supplementary Tables
