# Supplementary material for: Construction of a novel cancer-associated fibroblast-related signature to predict clinical outcome and immune response in colon adenocarcinoma
Source: Aging (Albany NY). 2023 Sep 16;15(18):9521–43. doi: 10.18632/aging.205032 (PMC10564434; doi:10.18632/aging.205032)
Supplement: Supplementary Figures [file aging-15-205032-s001.pdf]

SUPPLEMENTARY FIGURES

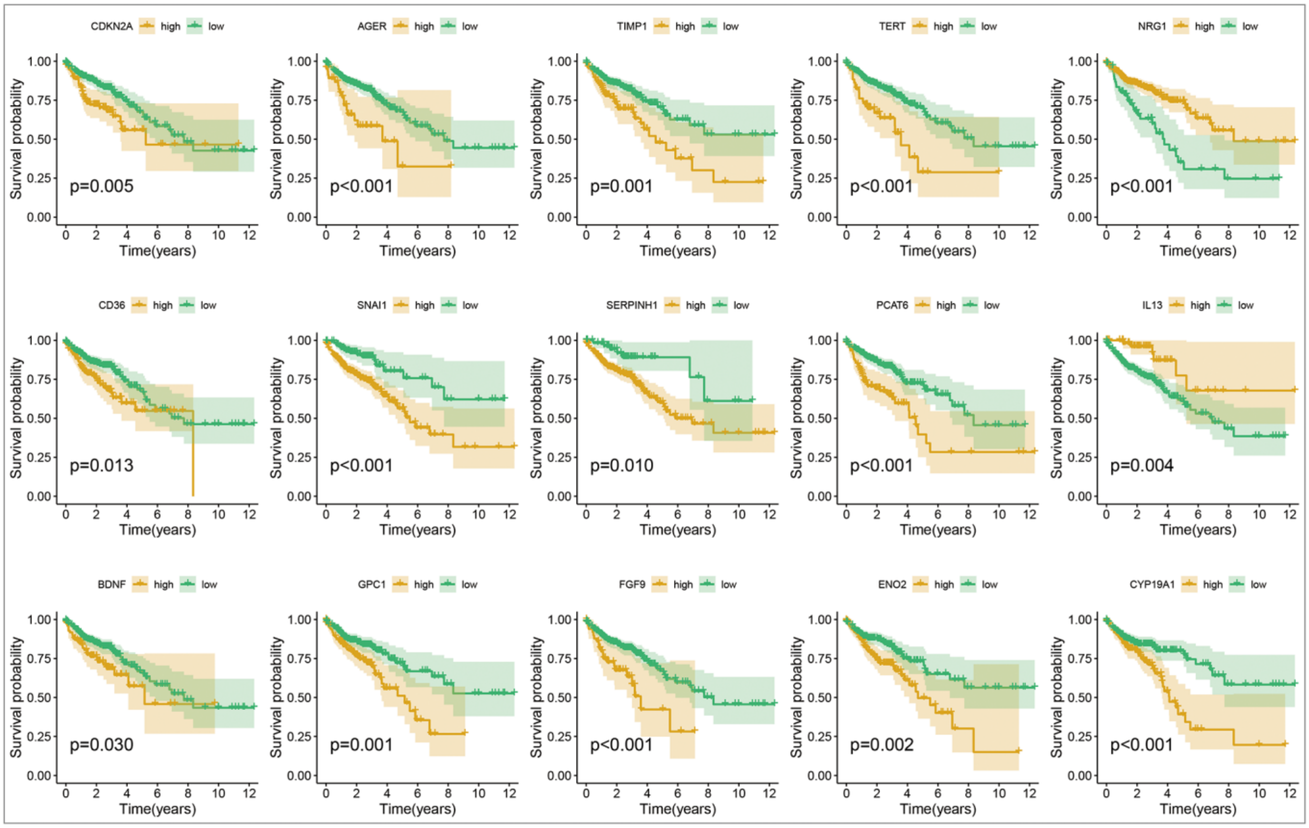

Supplementary Figure 1. K-M curves of signature-related genes in the TCGA-COAD cohort.

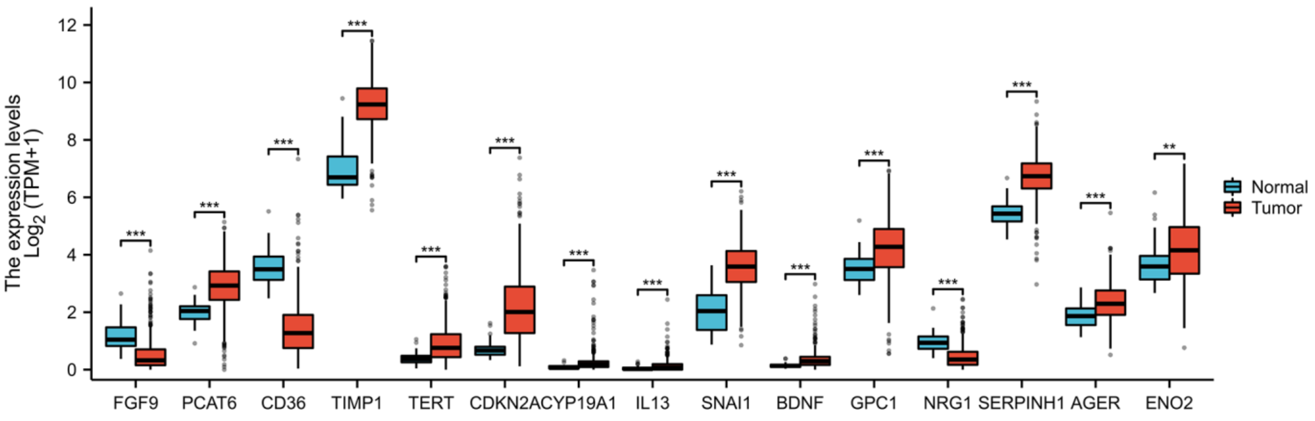

Supplementary Figure 2. The differential expression status of CAFRs-related genes in COAD tumor tissues and normal tissues in TCGA-COAD cohort.
