# Supplementary material for: Construction of a novel cancer-associated fibroblast-related signature to predict clinical outcome and immune response in colon adenocarcinoma
Source: Aging (Albany NY). 2023 Sep 16;15(18):9521–43. doi: 10.18632/aging.205032 (PMC10564434; doi:10.18632/aging.205032)
Supplement: Supplementary Tables [file aging-15-205032-s002.pdf]

SUPPLEMENTARY TABLES

Supplementary Table 1. Specific links to all immunohistochemistry images from the Human Protein Atlas.

Protein	Specific link
FGF9	Normal: https://www.proteinatlas.org/ENSG00000102678-FGF9/tissue/colon<br>Tumor: https://www.proteinatlas.org/ENSG00000102678-FGF9/pathology/colorectal+cancer#ihc
CD36	Normal: https://www.proteinatlas.org/ENSG00000135218-CD36/tissue/colon<br>Tumor: https://www.proteinatlas.org/ENSG00000135218-CD36/pathology/colorectal+cancer#img
TIMP1	Normal: https://www.proteinatlas.org/ENSG00000102265-TIMP1/tissue/colon<br>Tumor: https://www.proteinatlas.org/ENSG00000102265-TIMP1/pathology/colorectal+cancer#img
TERT	Normal: https://www.proteinatlas.org/ENSG00000164362-TERT/tissue/colon#img<br>Tumor: https://www.proteinatlas.org/ENSG00000164362-TERT/pathology/colorectal+cancer#img
CDKN2A	Normal: https://www.proteinatlas.org/ENSG00000147889-CDKN2A/tissue/colon#img<br>Tumor: https://www.proteinatlas.org/ENSG00000147889-CDKN2A/pathology/colorectal+cancer#img
CYP19A1	Normal: https://www.proteinatlas.org/ENSG00000137869-CYP19A1/tissue/colon#img<br>Tumor: https://www.proteinatlas.org/ENSG00000137869-CYP19A1/pathology/colorectal+cancer#img
SNAI1	Normal: https://www.proteinatlas.org/ENSG00000124216-SNAI1/tissue/colon#img<br>Tumor: https://www.proteinatlas.org/ENSG00000124216-SNAI1/pathology/colorectal+cancer#img
BDNF	Normal: https://www.proteinatlas.org/ENSG00000176697-BDNF/tissue/colon#img<br>Tumor: https://www.proteinatlas.org/ENSG00000176697-BDNF/pathology/colorectal+cancer#img
GPC1	Normal: https://www.proteinatlas.org/ENSG00000063660-GPC1/tissue/colon#img<br>Tumor: https://www.proteinatlas.org/ENSG00000063660-GPC1/pathology/colorectal+cancer#img
NRG1	Normal: https://www.proteinatlas.org/ENSG00000157168-NRG1/tissue/colon#img<br>Tumor: https://www.proteinatlas.org/ENSG00000157168-NRG1/pathology/colorectal+cancer#img
SERPINH1	Normal: https://www.proteinatlas.org/ENSG00000149257-SERPINH1/tissue/colon#img<br>Tumor: https://www.proteinatlas.org/ENSG00000149257-SERPINH1/pathology/colorectal+cancer#img
AGER	Normal: https://www.proteinatlas.org/ENSG00000204305-AGER/tissue/colon#img<br>Tumor: https://www.proteinatlas.org/ENSG00000204305-AGER/pathology/colorectal+cancer#img
ENO2	Normal: https://www.proteinatlas.org/ENSG00000111674-ENO2/tissue/colon#img<br>Tumor: https://www.proteinatlas.org/ENSG00000111674-ENO2/pathology/colorectal+cancer#img

Supplementary Table 2. Primer sequences used for RT-qPCR.

Gene	Primer sequence
GAPDH	F: ACAACTTTGGTATCGTGGAAGG<br>R: GCCATCACGCCACAGTTTC
ENO2	F: CCGGGAACTCAGACCTCATC<br>R: CTCTGCACCTAGTCGCATGG
FGF9	F: ATGGCTCCCTTAGGTGAAGTT<br>R: CCCAGGTGGTCACTTAACAAAAC
GPC1	F: TGAAGCTGGTCTACTGTGCTC<br>R: CCCAGAACTTGTCGGTGATGA
CD36	F: CTTTGGCTTAATGAGACTGGGAC<br>R: GCAACAAACATCACCACACCA
TIMP1	F: ACCACCTTATACCAGCGTTATGA<br>R: GGTGTAGACGAACCGGATGTC
TERT	F: TCACGGAGACCACGTTTCAAA<br>R: TTCAAGTGCTGTCTGATTCCAAT

CDKN2A	F: ATGGAGCCTTCGGCTGACT<br>R: GTAACTATTCGGTGCGTTGGG
CYP19A1	F: ACTACAACCGGGTATATGGAGAA<br>R: TCGAGAGCTGTAATGATTGTGC
IL-13	F: CCTCATGGCGCTTTTGTTGAC<br>R: TCTGGTTCTGGGTGATGTTGA
SNAI1	F: TCGGAAGCCTAACTACAGCGA<br>R: AGATGAGCATTGGCAGCGAG
BDNF	F: TAACGGCGGCAGACAAAAAGA<br>R: TGCACTTGGTCTCGTAGAAGTAT
PCAT6	F: CCCCTCCTTACTCTTGGACAAC<br>R: GACCGAATGAGGATGGAGACAC
NRG1	F: TCCCATTAGAATATCAGTATCCACAG<br>R: CATAAGCGACACACACAGGATTTC
SERPINH1	F: GCGGGCTAAGAGTAGAATCG<br>R: ATGGCCAGGAAGTGGTTTG
AGER	F: ACTACCGAGTCCGTGTCTACC<br>R: GGAACACCAGCCGTGAGTT
